# Supplementary material for: Probing chemotaxis activity in Escherichia coli using fluorescent protein fusions
Source: Sci Rep. 2019 Mar 7;9:3845. doi: 10.1038/s41598-019-40655-x (PMC6405996; doi:10.1038/s41598-019-40655-x)
Supplement: Supplementary file 1 — Supplementary information [file 41598_2019_40655_MOESM1_ESM.pdf]

SUPPLEMENTAL MATERIAL to:

Probing chemotaxis activity in *Escherichia coli* using fluorescent protein fusions

Clémence Roggo, Nicolas Carraro and Jan Roelof van der Meer*

Department of Fundamental Microbiology, University of Lausanne, 1015 Lausanne, Switzerland

*Corresponding author

J. R. van der Meer

Department of Fundamental Microbiology

University of Lausanne

Bâtiment Biophore

Quartier UNIL-Sorge

1015 Lausanne, Switzerland

Tel. +41 21 692 5630

Email : janroelof.vandermeer@unil.ch

Figures S1-S5

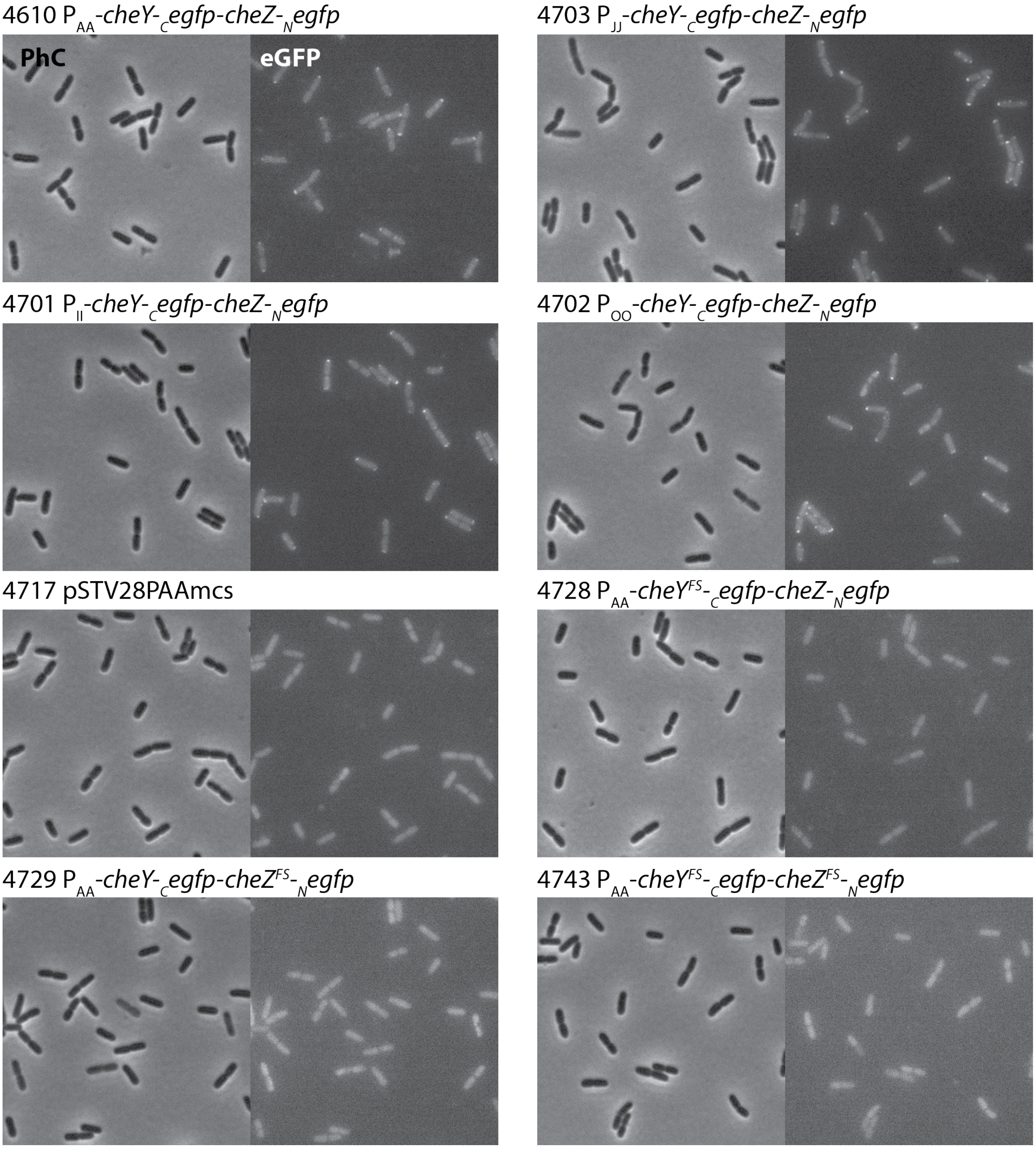

Figure S1: Microscopy images of split-eGFP strains. Phase contrast and GFP fluorescence images at 1000-fold magnification of *E. coli* expressing CheY or CheZ protein fusion with split-eGFP. (A)–(D) Strains carrying plasmids expressing *cheY*-$_{C}$*egfp*-*cheZ*-$_{N}$*egfp* under different promoter strengths (P_{AA}, P_{JJ}, P_{II}, P_{OO}), with clear foci visible. (E) *E. coli* transformed with the empty vector pSTV28PAAmcs. (F)–(H) *E. coli* with frameshift mutations in the plasmid-located genes *cheY* (4728), *cheZ* (4729) or in both *cheY* and *cheZ* (4743), abolishing the formation of foci.

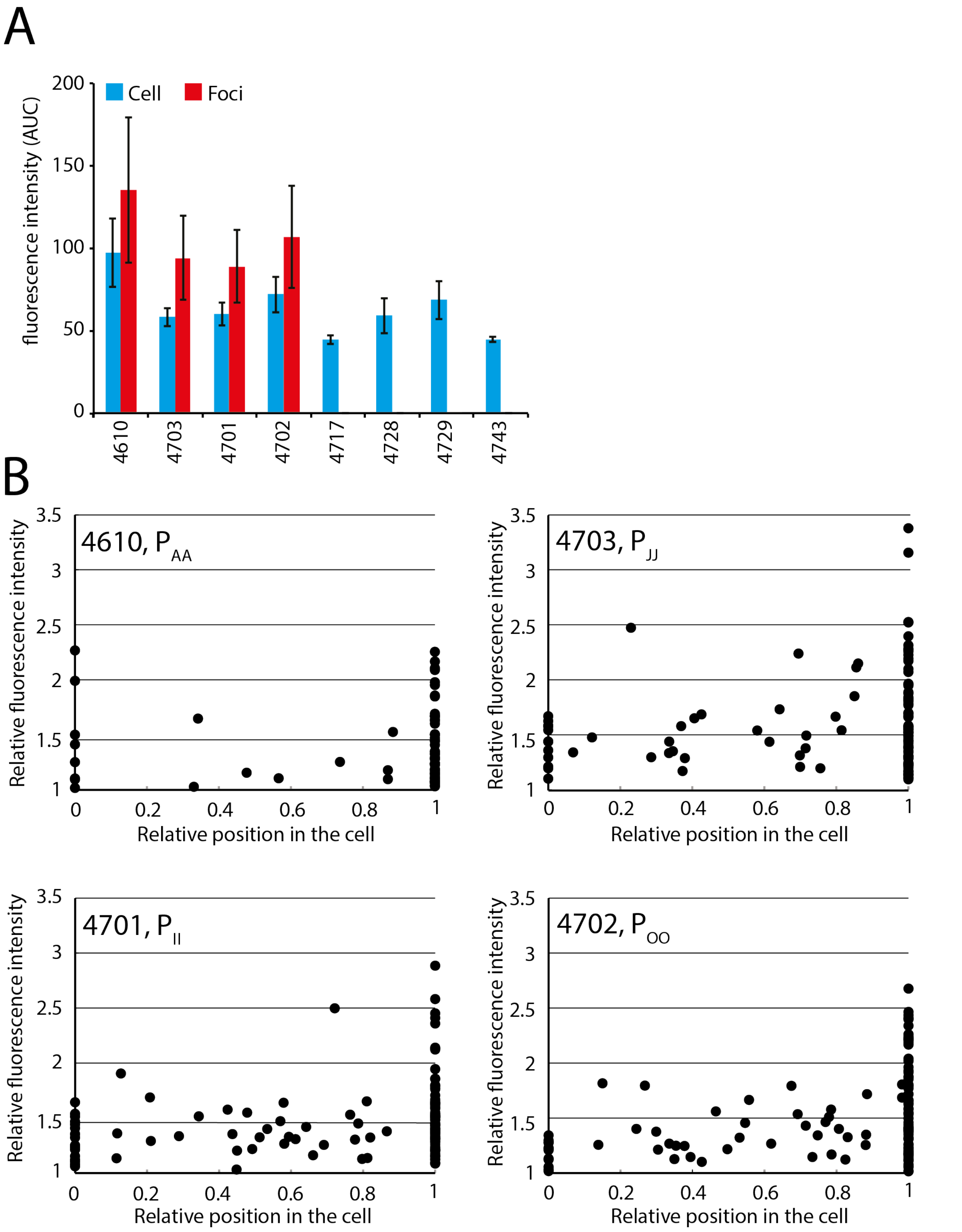

Figure S2: Fluorescent foci quantification. (A) Average of fluorescence intensity across the whole cell and in individual foci. **(B)** Foci localization and their relative fluorescence intensity along individual cells. The relative fluorescence intensity was calculated by dividing the fluorescence intensity of an individual focus by the average fluorescence of its cell. Localization was normalized by the length of the individual cell (0 and 1 correspond to cell poles). Strain numbers correspond to Table 1.

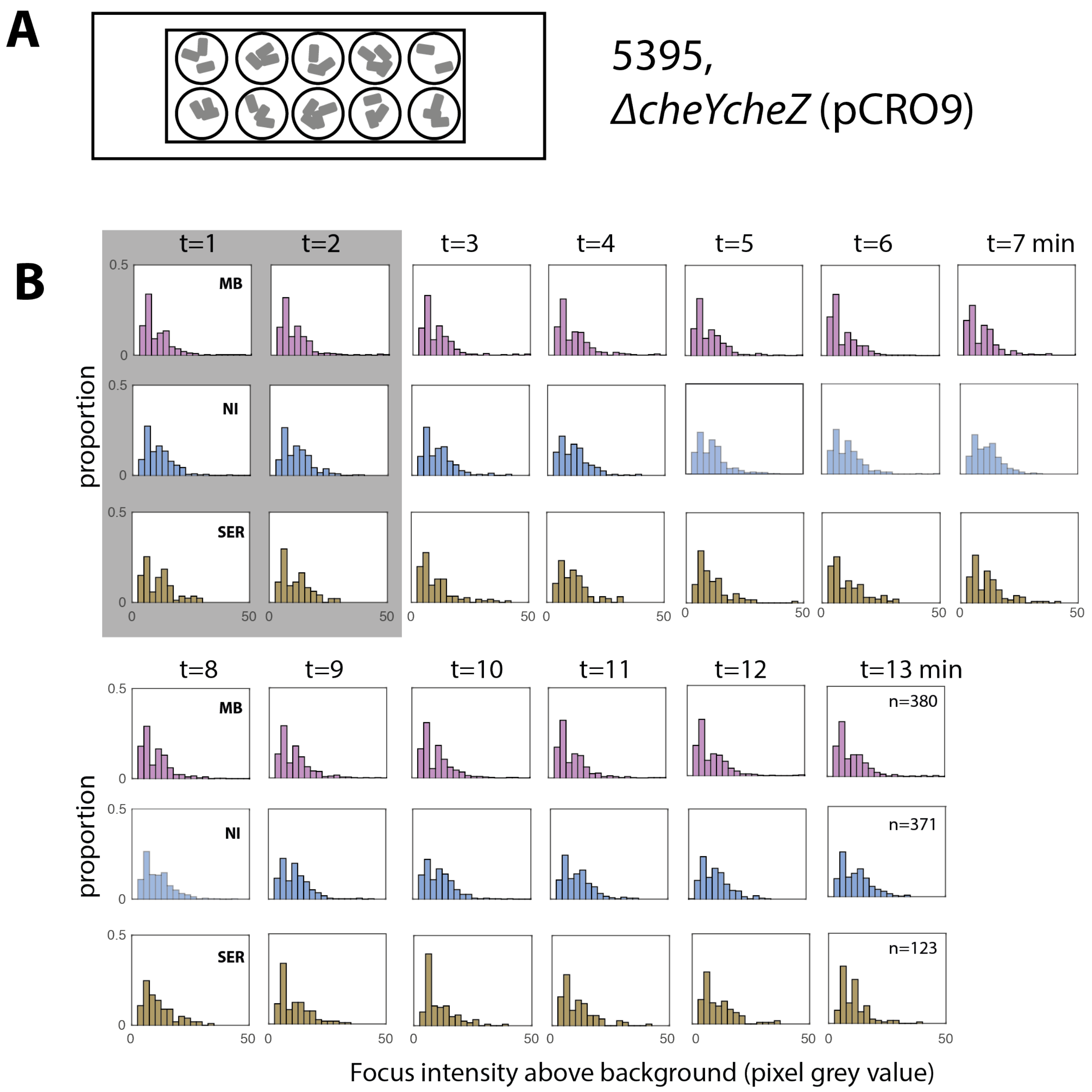

Figure S3: Foci intensity distributions among surface-adhered *E. coli* 5395 cells exposed in wells to motility buffer (MB), 100 µM $NiCl_2$ or 100 µM serine. (A) Schematic test outline. (B) Binned distributions of foci as a function of intensity, filtered for a background focus score = 3, imaged every minute. Addition of attractant or repellent after 2 minutes. n, number of cells.

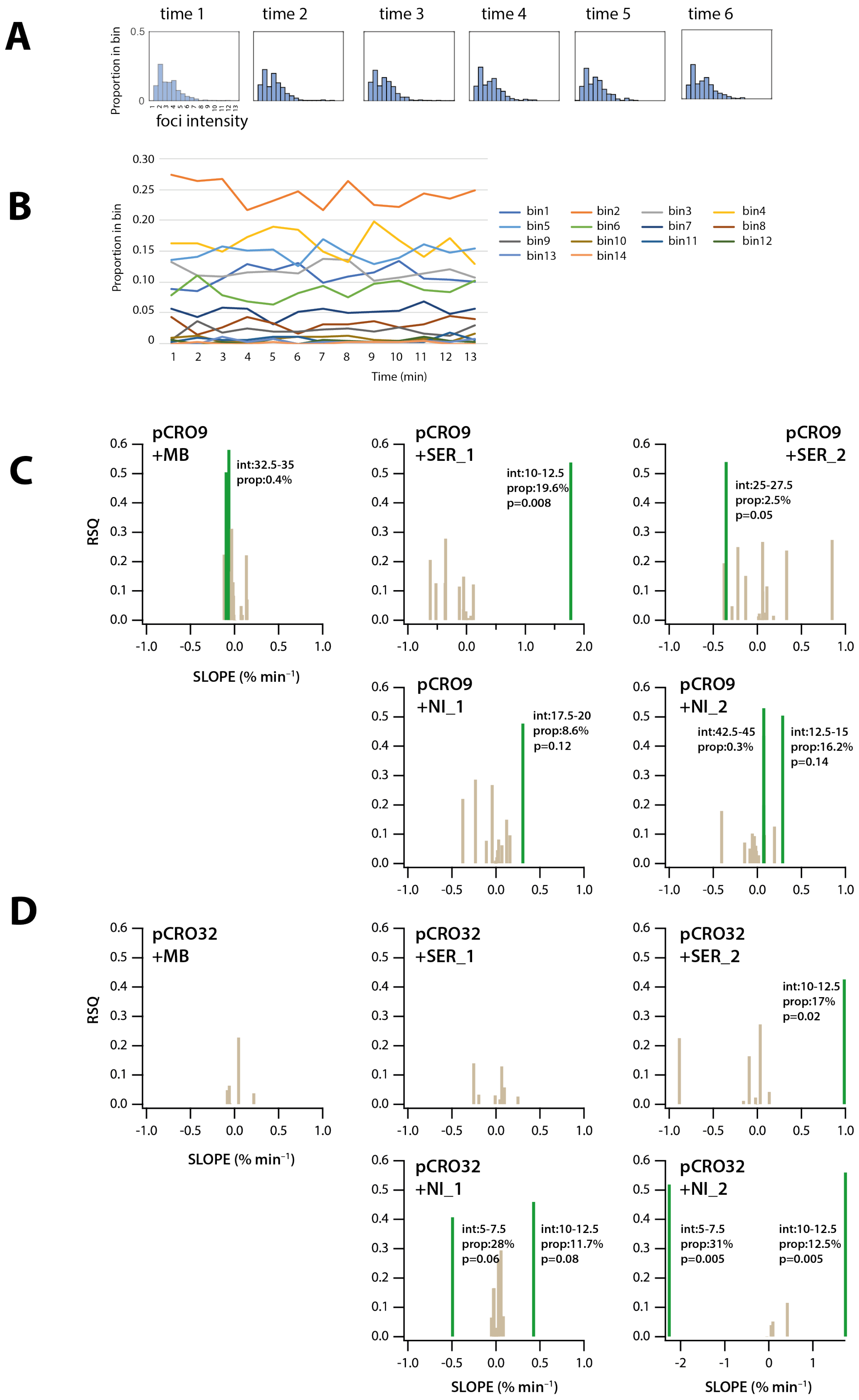

A
time 1
time 2
time 3
time 4
time 5
time 6
Proportion in bin
0.5
0
foci intensity
B
Proportion in bin
0.30
0.25
0.20
0.15
0.10
0.05
0
Time (min)
bin1
bin2
bin3
bin4
bin5
bin6
bin7
bin8
bin9
bin10
bin11
bin12
bin13
bin14
C
pCRO9 +MB
int:32.5-35
prop:0.4%
RSQ
SLOPE (% min⁻¹)
pCRO9 +SER_1
int:10-12.5
prop:19.6%
p=0.008
pCRO9 +SER_2
int:25-27.5
prop:2.5%
p=0.05
pCRO9 +NI_1
int:17.5-20
prop:8.6%
p=0.12
pCRO9 +NI_2
int:42.5-45
prop:0.3%
int:12.5-15
prop:16.2%
p=0.14
D
pCRO32 +MB
RSQ
SLOPE (% min⁻¹)
pCRO32 +SER_1
pCRO32 +SER_2
int:10-12.5
prop:17%
p=0.02
pCRO32 +NI_1
int:5-7.5
prop:28%
p=0.06
int:10-12.5
prop:11.7%
p=0.08
SLOPE (% min⁻¹)
pCRO32 +NI_2
int:5-7.5
prop:31%
p=0.005
int:10-12.5
prop:12.5%
p=0.005
SLOPE (% min⁻¹)

Figure S4: Dynamic changes of foci fluorescence among surface-adhered *E. coli* cells expressing stable or unstable split-eGFP. (A) Example foci intensity distribution over time (as in Figure S2). (B) Foci proportions are plotted per intensity bin of (A) over all time points, after which slopes of the trend line are calculated by linear regression. (C) Plot of the per-bin regression lines of calculated *slope* versus r^2 (RSQ). Note that slopes close to 0 are neither increasing or decreasing. RSQ-values above 0.4 were considered presenting significant trends and are shown in green. Slopes with RSQ >0.4 have indicated the *proportion* of that bin-category of all foci, the *intensity level* of that bin and the calculated p-value for the slope being different from 0. For example, a positive slope thus indicates an increase in that category over time; a negative slope a decrease over time.

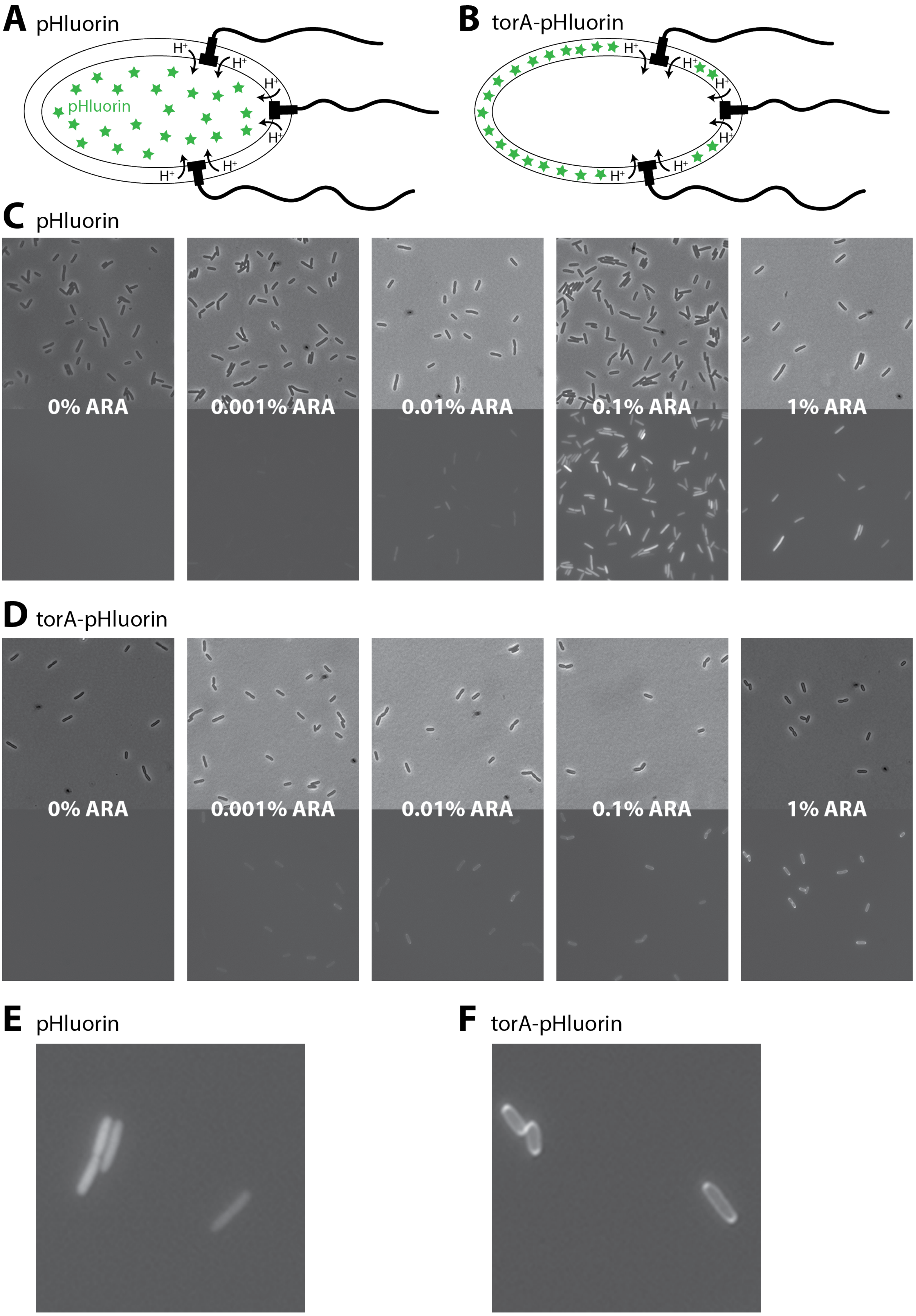

Figure S5: Induction of pHluorin expression in the *E. coli* cytoplasm and periplasm. Schematics of expected localization of for pHluorin without any tags in the cytoplasm (A)

and in the periplasm in case of attachment of the torA-signal sequence. (B) Phase contrast and epifluorescence images of cells incubated with tenfold increasing arabinose concentrations, for the case of cytoplasmic pHluorin (B) or periplasmic torA-pHluorin (C). Enlarged images of individual cells expressing pHluorin (E) or exporting the torA-pHluorin into the periplasm (F), after induction with 0.1 or 1% (*w/*v) arabinose, respectively.
